# Faster age-related decline in cardiorespiratory fitness in rheumatoid arthritis patients: an observational study in the Trøndelag Health Study

**DOI:** 10.1007/s00296-020-04713-2

**Published:** 2020-10-09

**Authors:** Marthe Halsan Liff, Mari Hoff, Ulrik Wisløff, Vibeke Videm

**Affiliations:** 1 Clinic of Orthopaedics, Rheumatology and Dermatology, St. Olavs University Hospital, Trondheim, Norway; 2 Department of Rheumatology, Levanger Hospital, Nord-Trøndelag Hospital Trust, Levanger, Norway; 3 Department of Clinical and Molecular Medicine, Lab Center 3 East, St. Olavs University Hospital, NTNU–Norwegian University of Science and Technology, 7006 Trondheim, Norway; 4 Department of Neuromedicine and Movement Science, NTNU–Norwegian University of Science and Technology, Trondheim, Norway; 5 Department of Public Health and Nursing, NTNU–Norwegian University of Science and Technology, Trondheim, Norway; 6 Department of Rheumatology, St. Olavs University Hospital, Trondheim, Norway; 7 Cardiac Exercise Research Group, Department of Circulation and Medical Imaging, NTNU–Norwegian University of Science and Technology, Trondheim, Norway; 8 School of Human Movement and Nutrition Sciences, University of Queensland, Brisbane, Australia; 9 Department of Immunology and Transfusion Medicine, St. Olavs University Hospital, Trondheim, Norway

**Keywords:** Cardiorespiratory fitness, Rheumatoid arthritis, Aging, Population-based study

## Abstract

Primary aim: Compare change in estimated cardiorespiratory fitness (eCRF change) in rheumatoid arthritis (RA) patients with population-based age- and sex-matched controls during ~ 11-year follow-up and identify variables associated with eCRF change. Secondary aim: Compare eCRF level in RA patients and controls. eCRF change from the second (HUNT2 1995–1997) to the third (HUNT3 2006–2008) surveys of the Norwegian Trøndelag Health Study was compared between RA patients (*n* = 188) and controls (*n* = 26,202) attending both surveys. Predictors of eCRF change were identified by Lasso regression followed by multiple linear regression. Mean eCRF level in RA patients (*n* = 436) and controls (*n* = 67,910) was compared using age-adjusted linear regression stratified on sex, as well as two-sample t tests including RA patients (*n* = 432) and controls (*n* = 59,124) who attended either HUNT2, HUNT3 or both HUNT2 and HUNT3. The mean eCRF decline from HUNT2 to HUNT3 in RA patients was 8.3 mL min^−1^ kg^−1^ versus 6.7 mL min^−1^ kg^−1^ in controls (*p* < 0.001). The decline was faster in RA patients and larger with higher baseline age (standardized regression coefficient for RA patients: (− 0.482 × age + 0.044); controls: (− 0.367 × age, *p* < 0.001). The decline was also associated with smoking, cardiovascular disease, increasing body mass index, asthma, and hypertension. Mean differences in age-adjusted eCRF level for RA patients versus controls (*p* < 0.001): women HUNT2: − 3.2 mL min^−1^ kg^−1^; HUNT3: − 5.0 mL min^−1^ kg^−1^; men HUNT2: − 1.8 mL min^−1^ kg^−1^; HUNT3: − 4.0 mL min^−1^ kg^−1^. Higher age at baseline was associated with faster decline in eCRF. This change was more pronounced in RA patients than controls, indicating a larger negative effect on fitness of aging in RA. RA patients had lower eCRF compared to healthy individuals.

**Electronic supplementary material:**

The online version of this article (10.1007/s00296-020-04713-2) contains supplementary material, which is available to authorized users.

## Introduction

Rheumatoid arthritis (RA) is an inflammatory disease of the joints [1], but it also affects internal organs, including the vasculature. RA patients are younger when they develop cardiovascular risk factors, suffer from more cardiovascular disease (CVD), and have higher mortality rates due to CVD than the general population [2–5]. Evidence supports that the chronic systemic inflammation associated with RA is an important driver of excess CVD in RA patients, particularly by causing accelerated atherosclerosis [6]. In addition, it has become evident that factors like reduced physical activity (PA) and increased levels of traditional risk factors for CVD contribute to the differences. PA affects cardiorespiratory fitness (CRF) [7, 8], and CRF is inversely associated with cardiovascular risk [7]. CRF is viewed as an independent risk factor for CVD and mortality [7, 9, 10], and has recently received much attention because it may be modified.

The gold standard method of measuring CRF is by testing maximum oxygen uptake during cardiopulmonary exercise testing (CPET), which is rather resource intensive [7]. For easier evaluation, equations for estimated cardiorespiratory fitness (eCRF) may be used, making it possible to investigate eCRF in big population-based studies without the need for a physical test [7]. eCRF equations are usually developed by multivariable regression analysis of variables expected to be associated with the maximum oxygen uptake measured by CPET, followed by removal of non-significant variables to achieve a simplified, yet appropriate regression model. Selected variables should be easily accessible, e.g., height, weight, waist circumference, resting heart rate (RHR) and/or answers to questionnaires describing PA habits. In this way, CRF may be calculated from the model with acceptable accuracy without performing CPET [11].

In the second and third surveys of the Norwegian population-based Trøndelag Health Study (HUNT2 and HUNT3) conducted in 1995–1997 and 2006–2008 [12], formulas for eCRF for healthy participants were developed [10, 11]. Using these eCRF equations, the associations of eCRF to various risk factors and outcomes have been investigated [10, 13, 14]. After demonstrating that these formulas overestimated eCRF in RA patients with the lowest measured CRF, our group developed eCRF equations that more correctly calculate eCRF in RA patients [15]. Previous studies suggest that RA patients are deconditioned and on average have decreased CRF compared to the general population [16–18]. To our knowledge, no studies have compared age-related changes in eCRF of RA patients and healthy people in a population-based setting. The design of the large population-based HUNT study with long follow-up makes this possible.

On this background, we hypothesized that eCRF in RA patients deteriorates faster by time compared to controls, and that RA patients in HUNT2 and HUNT3 are deconditioned and have lower eCRF than controls. Thus, the primary aim of the present study was to investigate the change of eCRF by time from HUNT2 to HUNT3 in RA patients compared to controls and identify variables associated with the potential difference in this change between the two groups. The secondary aim was to compare eCRF levels between RA patients and controls in HUNT2 and HUNT3.

## Methods

The present work was a sub-study of HuLARS (HUNT Longitudinal Ankylosing spondylitis and Rheumatoid Arthritis Study). In the HUNT study [12], all inhabitants ≥ 20 years old from the northern part of the Norwegian county of Trøndelag were invited. The HUNT study is an open cohort study and data, including results from questionnaires and blood samples from participants from HUNT2 (1995–1997) and HUNT3 (2006–2008), were used in the present observational study.

Power was calculated based on the following assumptions using data from previous HUNT publications [19, 20]: Approximately 33,000 persons participated in both HUNT2 and HUNT3 and the prevalence of RA was ~ 0.75%; we expected ~ 15% missing data for calculation of eCRF; the average 10-year decline in CRF in healthy people would be ~ 3.8 mL min^−1^ kg^−1^; we presumed a 20% larger decline in individuals with RA; and used alpha = 0.05 and a two-sided test. The calculated power was 82%, which was considered sufficient to perform the study.

### Patients

**Fig. 1:**
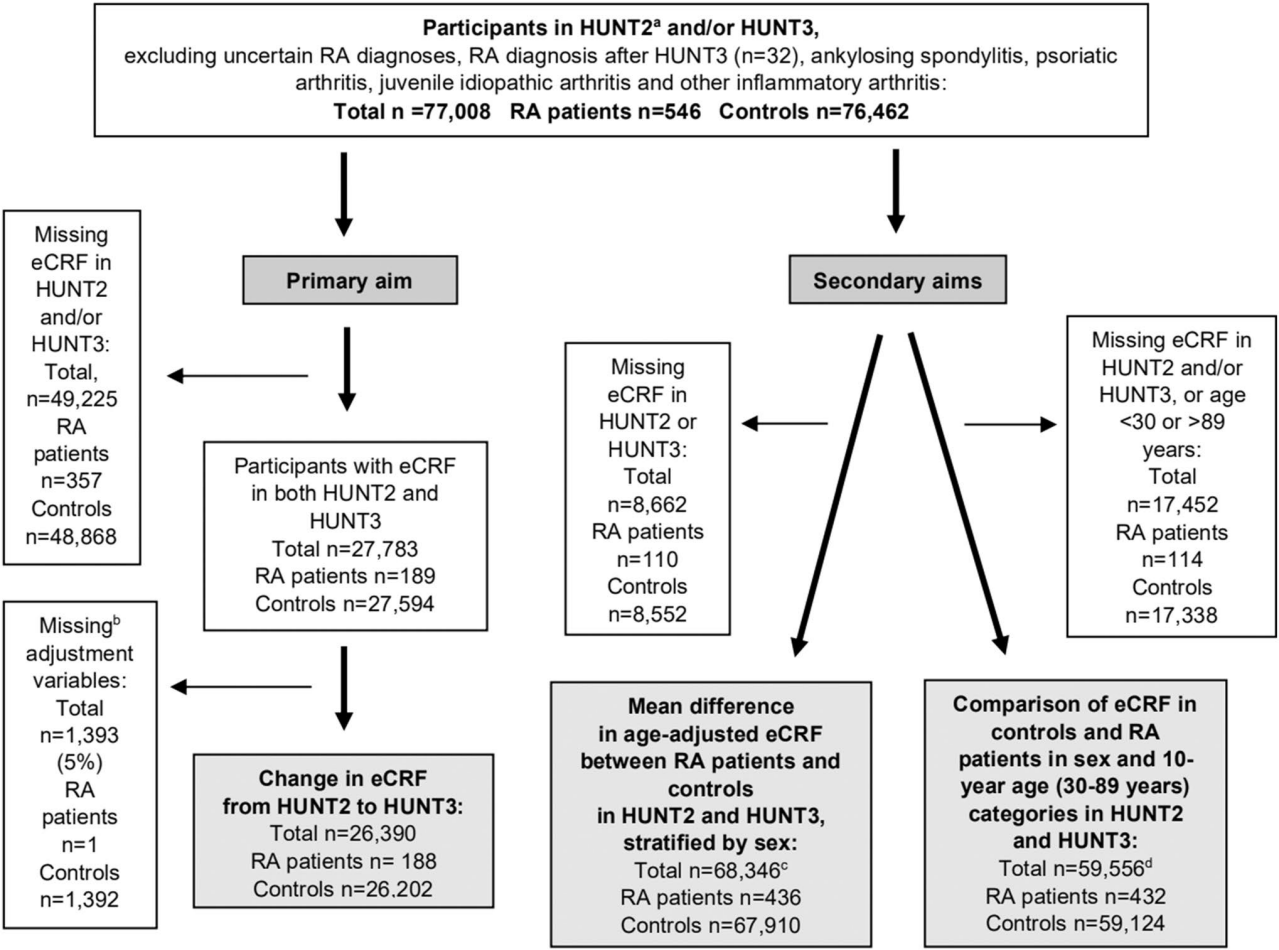
Recruitment to the study. **a** *HUNT2 and HUNT3* Second and third surveys of the Trøndelag Health Study, *RA* rheumatoid arthritis, *eCRF* estimated cardiorespiratory fitness, *HUNT3 Fitness* Sub-study of HUNT3. **b** Missing data: < 0.1% for body mass index and asthma, < 0.2% for cardiovascular disease and hypertension, and 4.5% for smoking. Remaining variables complete. **c** Numbers represent unique participants. Because persons participating both in HUNT2 and HUNT3 were included in the analysis for both timepoints, the actual n was higher (total *n* = 96,129, RA patients *n* = 625, controls *n* = 95,504). **d** Numbers represent unique participants. Because persons participating both in HUNT2 and HUNT3 were included in the analysis for both timepoints, the actual n was higher (total *n* = 84,170, RA patients *n* = 616, controls *n* = 83,554)

Based on the information in hospital case files and using the standardized 2010 ACR/European League Against Rheumatism classification criteria for rheumatoid arthritis [20–22] or for some cases diagnosed before 2010 the 1987 American College of Rheumatology (ACR) classification criteria due to insufficient information [21], a previous study identified those with a valid RA diagnosis (*n* = 578) out of all participants in HUNT2 and HUNT3 who self-reported RA. We excluded those who received an RA diagnosis after HUNT3 (*n* = 32) and participants with ankylosing spondylitis, psoriasis arthritis, juvenile idiopathic arthritis, or other inflammatory arthritis. The remaining participants were included as controls. The primary aim was to investigate the change in eCRF from HUNT2 to HUNT3; thus, we only included controls and RA patients with valid eCRF in both HUNT2 and HUNT3 and with no missing adjustment variables in the regression analysis (188 RA patients and 26,202 controls) in this analysis (Fig. 1). For the secondary aims comparing eCRF levels in controls and RA patients, we included participants attending either HUNT2 only, HUNT3 only, or HUNT2 and HUNT3, resulting in a higher number of participants for these comparisons as detailed in Fig. 1. Method validation was performed in participant subsets as further described below.

### Main outcome variable

eCRF (mL kg^−1^ min^−1^) was calculated using the previously published eCRF equations for healthy controls [10, 11], and the RA-specific equation for RA patients [15]. Due to collinearity, variables present in eCRF equations cannot be used as explanatory variables for eCRF in a novel regression analysis. This problem was avoided for the primary aim in the present study because the outcome variable was the change in eCRF from HUNT2 to HUNT3.

### Study factors

The primary and secondary outcomes were compared among RA patients and controls as defined above.

### Other variables

Variables known from the literature to be associated with eCRF change and available in the HUNT surveys were used. The following variables and definitions were used: CVD (yes/no)—self-reported prior or present angina pectoris and/or myocardial infarction and/or stroke. Family CVD history (yes/no)—previous/present stroke and/or hypertension and/or myocardial infarction (MI) before age 60 years in a first-degree relative. Hypertension (yes/no)—blood pressure ≥ 140/90 mm Hg and/or self-reported use of anti-hypertensive medication. Hypertension and systolic blood pressure (SBP) are correlated, and only hypertension was used because those treated with anti-hypertensive medication might have normalized SBP despite a diagnosis of hypertension. Smoking (yes/no)—self-reported prior or present smoking. Asthma (yes/no)—self-reported prior or present asthma. Diabetes (yes/no)—self-reported diabetes and/or the use of anti-diabetic medication and/or having a non-fasting blood-glucose level > 11 mmol × L^−1^. Cancer (yes/no)—self-reported prior or present cancer. Pain (yes/no)—pain and/or stiffness that had lasted for ≥ 3 of the 12 latest months. Body mass index—weight/squared height (kg/m^2^). High-density lipoprotein (HDL) cholesterol measured in mmol/L.

PA strongly influences CRF [7, 8]. The American College of Sports Medicine and American Heart Association’s (ACSM/AHA) recommendations for aerobic PA are to perform either moderate-intensity physical activity ≥ 30 min on ≥ 5 days each week (≥ 150 min per week) or to perform vigorous-intensity aerobic activity ≥ 20 min ≥ 3 days a week (≥ 75 min per week). PA at these two intensities may also be combined [10, 23]. To describe the level of PA, the proportions of RA patients and controls fulfilling the ACSM/AHA recommendations for aerobic PA at HUNT2 (baseline) and HUNT3 were calculated from responses to questions about frequency, intensity and duration of weekly performed PA [10, 11, 23].

### Ethics statement

All participants in HUNT2 and HUNT3 provided written informed consent. The present study was approved by The Regional Committee for Medical and Health Research Ethics (4.2009.1068 and 2018/1149) and was performed in compliance with the Helsinki Declaration.

### Statistical analysis

Data are given as counts or mean with percentages or standard deviation (SD) in parenthesis. *p* values < 0.05 were considered significant. Analyses were performed using STATA (Version 15.0, StataCorp, College Station, TX, USA).

To evaluate the decline in eCRF from HUNT2 to HUNT3 for the primary aim, regression models were performed in steps with different adjustments. In Step 1, we performed multiple linear regression with change in eCRF as the dependent variable and age (continuous), RA status (yes/no), and the interaction term for age and RA status as independent variables, which permitted investigation of whether eCRF reduction by time was different between RA patients and controls depending on age. Inclusion of age in the model ensured that differences in baseline age between RA patients and controls were adjusted for. We also included the following predefined adjustment variables: baseline eCRF, sex (male = 0 and female = 1), and time from participation in HUNT2 to participation in HUNT3 (years). Baseline eCRF, sex and age were included because the change in eCRF may depend on the starting level, and CRF varies with sex and age. Adjustment for time between the HUNT2 and HUNT3 was included because time varied from 10 to 12 years among individual participants.

The Step 1 model was then further modified to investigate other associations to the decline in eCRF from HUNT2 to HUNT3 (Step 2–4). Based upon literature, further baseline variables possibly relevant for the change in eCRF were considered as detailed above (CVD, family CVD history, hypertension, smoking, asthma, diabetes, cancer, pain, BMI, and HDL cholesterol). PA and RHR could not be included in the main analysis of change of eCRF because of collinearity with the dependent variable.

To reduce the risk of overfitting and promote reliable variable selection, the mentioned explanatory variables were first analyzed by Lasso (least absolute shrinkage and selection operator) regression (*n* = 1000 repetitions). Lasso identifies the smallest useful set of variables among variables that may be highly correlated, and gives irrelevant variables a coefficient of 0 [24]. Variables with a coefficient different from 0 in the Lasso regression were, therefore, added to the Step 1 model to achieve the Step 2 model. The Step 2 model was then reduced to the final Step 3 model by removal of non-significant variables. In Step 4, the Step 3 model was standardized to compare the effect sizes of the predictors.

The models were compared using the *R*^2^ (i.e., the variation in the dependent variable explained by the independent variables), root mean square error (RMSE, i.e., standard error of the residuals, which tells how close the data lie around the line of best fit), Akaike information criterion (AIC) and Bayesian information criterion (BIC), where low numbers mean that the model better fits the data. Assumptions were evaluated using residual plots.

For the secondary aims, analysis was performed separately for HUNT2 and HUNT3 and each participant was included wherever she/he had participated (Fig. 1). Linear regression was used to find the mean sex-specific age-adjusted difference in eCRF between RA patients and controls. Mean eCRF of controls and RA patients aged 30–89 years were further compared with two-sample *t* tests in ten-year age categories for each sex separately.

As a sensitivity assay, we validated whether the eCRF calculation methods used in the study were comparable employing equivalence testing. With this method, the mean and 90% confidence interval (CI) of the difference between two methods, e.g., the calculated eCRF and measured CRF are evaluated against a predefined equivalence region [25]. The equivalence region indicates how big the difference may be for the two methods still to be considered equivalent. As there is no generally accepted equivalence region for eCRF vs. measured CRF, we evaluated against an equivalence region of ± 1 Metabolic Equivalent (MET) (± 3.5 mL min^−1^ kg^−1^).

The eCRF equation for the general population was developed from a sub-study of HUNT3 (HUNT3 Fitness) [11, 19], which ensures that the eCRF equation for the general population fits the controls of our study. To evaluate whether the RA-specific eCRF equation would be adequate for the controls, an equivalence test was performed to compare the calculated eCRF by the RA-specific equation to the measured CRF from CPET in 3,294 of the controls in our study (women, *n* = 1754 and men, *n* = 1540), who had also participated in the HUNT3 Fitness study.

The equations for estimation of the RA-specific eCRF in HUNT2 and HUNT3 were slightly different due to the registered variables concerning PA in each survey. In a second equivalence test, we, therefore, compared these two RA equations in 189 RA patients where data for both methods were available. There are similar differences in the eCRF equations used in controls in HUNT2 and HUNT3. Thus, a third equivalence test of the general eCRF equations for HUNT2 and HUNT3 in 27,594 controls was also performed.

## Results

Baseline characteristics, including mean eCRF in HUNT2 and the frequencies of RA patients and controls fulfilling the ACSM/AHA recommendation for aerobic PA at baseline are given in Table 1. Table 1 presents baseline characteristics for RA patients (*n* = 188) and controls (*n* = 26,202), after exclusion of those with missing data for variables in the main regression analysis of change of eCRF. In HUNT2, 48% of the women with RA and 58% of the control women fulfilled aerobic PA recommendations, and the corresponding figures for men were 61% and 66%, respectively. In HUNT3, 31% of the women with RA and 40% of the control women fulfilled aerobic PA recommendations, and the corresponding figures for men were 29% and 41%, respectively.

**Table 1:** Baseline characteristics for the main analysis

Total, *n* = 26,390	Women			Men		
	Controls *n* = 14,466	RA patients *n* = 119	*p* value	Controls *n* = 11,736	RA patients *n* = 69	*p* value
Age, mean (SD) (years)	44.9 (12.8)	52.4 (10.5)	< 0.001	46.8 (12.7)	55.6 (9.7)	< 0.001
Systolic blood pressure, mean (SD) (mm Hg)	129.6 (19.0)	134.0 (16.4)	0.01	137.0 (16.4)	141.0 (19.8)	0.06
Resting heart rate, mean (SD) (bpm)	72.2 (12.2)	73.1 (10.8)	0.42	67.0 (12.3)	68.4 (12.7)	0.33
Body mass index, mean (SD) (kg/m^2^)	25.6 (4.1)	26.7 (4.0)	0.006	26.4 (3.2)	26.3 (3.3)	1.00
Waist circumference, mean (SD) (cm)	79.4 (10.3)	83.0 (10.6)	< 0.001	91.0 (8.4)	91.6 (9.2)	0.57
High-density lipoprotein mean (SD) (mmol/L)	1.53 (0.38)	1.50 (0.45)	0.42	1.25 (0.33)	1.23 (0.33)	0.53
Ever smoker, *n* (%)	7191 (50)	69 (58)	0.07	6359 (54)	48 (70)	0.01
Cardiovascular disease^a^, *n* (%)	237 (2)	3 (3)	0.45	575 (5)	8 (12)	0.01
Asthma^b^, *n* (%)	1084 (8)	9 (8)	1.00	933 (8)	3 (4)	0.27
Hypertension^c^, *n* (%)	3994 (28)	46 (39)	0.01	4923 (42)	39 (57)	0.02
Diabetes^d^, *n* (%)	144 (1)	3 (3)	0.10	183 (2)	4 (6)	0.005
Fulfills ACSM/AHA recommendations for aerobic PA, *n* (%)	8522 (59)	57 (48)	0.02	7768 (66)	42 (61)	0.35
eCRF in HUNT2, mean (SD) (mL min^−1^ kg^−1^)	36.81 (5.8)	31.19 (6.2)	< 0.001	46.10 (6.8)	40.95 (8.2)	< 0.001

*RA* rheumatoid arthritis, *bpm* beats per minute, *ACSM* American College of Sports Medicine; *AHA* American Heart Association, *PA* physical activity, *eCRF* estimated cardiorespiratory fitness, *HUNT2* The second survey of the Trøndelag Health Study

^a^Cardiovascular disease: Self-reported prior or present angina pectoris and/or myocardial infarction and/or stroke

^b^Asthma: Self-reported prior or present asthma

^c^Hypertension: Blood pressure ≥ 140/90 and/or self-reported use of anti-hypertensive medication

^d^Diabetes: Self-reported diabetes and/or the use of anti-diabetic medication and/or having a non-fasting blood-glucose level > 11 mmol × L^−1^

### Primary aim

The mean change in eCRF from HUNT2 to HUNT3 was − 8.3 mL min^−1^ kg^−1^ in RA patients compared to − 6.7 mL min^−1^ kg^−1^ in controls (*p* < 0.001); for women: − 7.5 (3.7) mL min^−1^ kg^−1^ for RA patients vs. − 6.0 (3.4) mL min^−1^ kg^−1^ for controls; for men: − 9.6 (3.3) mL min^−1^ kg^−1^ in RA patients vs. − 7.6 (4.1) mL min^−1^ kg^−1^ for controls.

**Fig. 2:**
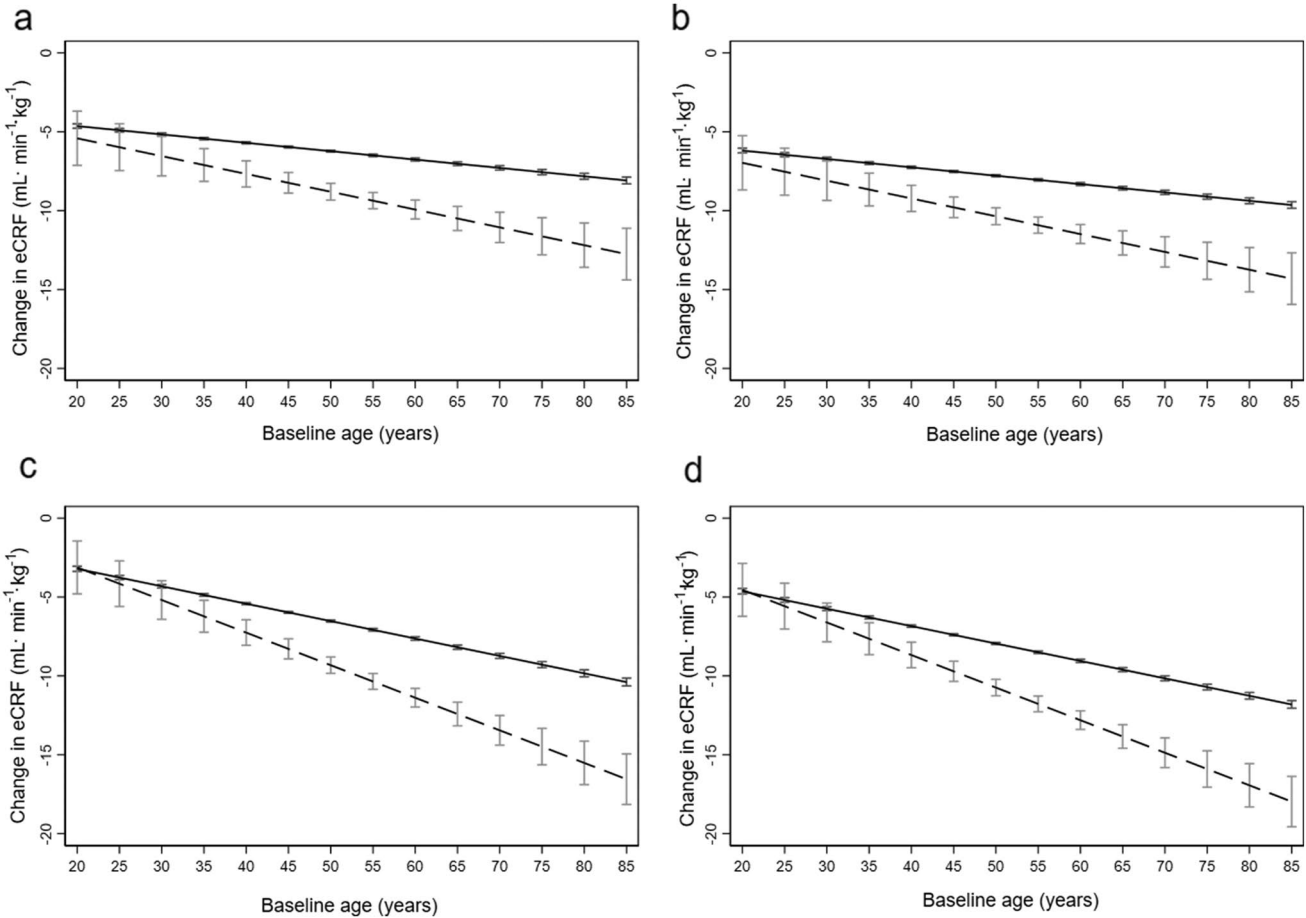
Change of eCRF^a^ from HUNT2 to HUNT3. Change of eCRF from HUNT2 to HUNT3 for RA patients (------) and controls (––––) with 95% confidence intervals. Panels **a** (women) and **b** (men) represent the Step 1 model including RA status (yes/no), age, and the interaction term for age and RA status with adjustment for baseline eCRF, sex and time between HUNT2 and HUNT3. Panels **c** (women) and **d** (men) represent the Step 3 model, additionally adjusted for smoking (never vs. ever), cardiovascular disease, body mass index, high-density lipoprotein, asthma and hypertension. ^a^*eCRF* estimated cardiorespiratory fitness, *HUNT2 and HUNT3* The second and third surveys of the Trøndelag Health Study, *RA* rheumatoid arthritis

The Step 1 regression model for change in eCRF from HUNT2 to HUNT3 showed that the decline was larger in RA patients compared to controls and increasing with older age at baseline (Table 2, Fig. 2, panel a and b). No potential adjustment variables had a coefficient of 0 in the Lasso regression, so all variables were included in the Step 2 model. Cancer, diabetes, pain, and family CVD history were non-significant in Model 2 and were removed from Model 3. Removal of these variables hardly influenced model fit. The adjustment provided by smoking, CVD, BMI, HDL cholesterol, asthma and hypertension in the Step 3 model (Table 2) rendered the decline in eCRF from HUNT2 to HUNT3 even more pronounced (Fig. 2, panel c and d). Based on the Step 4 model, the age-related eCRF decrease in RA patients was (− 0.482 × age + 0.044) mL min^−1^ kg^−1^ compared to (− 0.367 × age) mL min^−1^ kg^−1^ in controls.

**Table 2:** Regression models for eCRF change (mL min^−1^ kg^−1^) with standardization

	Step 1 model^a^	Step 2 model^b^	Step 3 model^c^	Step 4 model^d^
Age (years)	− 0.053***	− 0.110***	− 0.110***	− 0.367
RA status (no = 0/yes = 1)	0.421 (*p* = 0.76)	2.138 (*p* = 0.12)	2.011 (*p* = 0.13)	0.044
Age and RA interaction	− 0.060*	− 0.101***	− 0.096***	− 0.115
Baseline eCRF (mL min^−1^ kg^−1^)	− 0.271***	− 0.475***	− 0.473***	− 0.964
Years from HUNT2 to HUNT3	− 0.3771***	− 0.334***	− 0.339***	− 0.050
Sex (male = 0/female = 1)	− 0.965***	− 3.361***	− 3.320***	− 0.431
Smoking (never = 0/ever = 1)		− 0.474***	− 0.518***	− 0.068
Cardiovascular disease (no = 0/yes = 1)		− 0.279*	− 0.339**	− 0.015
Body mass index (kg/(m^2^))		− 0.286***	− 0.292***	− 0.285
High-density lipoprotein concentration		0.336***	0.289***	0.029
Asthma (no = 0/yes = 1)		− 0.253*	− 0.216**	− 0.015
Hypertension (no = 0/yes = 1)		− 0.277***	− 0.211***	− 0.026
Pain (no = 0/yes = 1)		0.0310 (*p* = 0.51)		
Cancer (no = 0/yes = 1)		0.0557 (*p* = 0.70)		
Diabetes (no = 0/yes = 1)		− 0.188 (*p* = 0.36)		
Family CVD history (no = 0/yes = 1)		0.010 (*p* = 0.83)		
Constant	11.561	30.706	30.893	
R squared	0.16	0.21	0.21	
RMSE	3.52	3.39	3.41	

The standardized coefficient gives the change in eCRF for one SD increase in each continuous variable, and the change in eCRF for the change from 0 to 1 in each categorical variable

*eCRF* estimated cardiorespiratory fitness, *RA* rheumatoid arthritis, *HUNT2 and HUNT3* The second and third surveys of the Trøndelag Health Study, *CVD* cardiovascular disease, *R−squared* the variation in the dependent variable explained by the independent variables, *RMSE* root mean square error; *Lasso* least absolute shrinkage and selection operator regression

*p < 0.05, **p < 0.01, ***p < 0.001

^a^After removal of variables because of collinearity and high number of missing

^b^After Lasso regression

^c^After removal of non-significant variables

^d^After standardization

### Secondary aims

eCRF in RA patients was lower than eCRF in controls. Mean differences in age-adjusted eCRF for RA patients versus controls were: women HUNT2: − 3.2 mL min^−1^ kg^−1^; women HUNT3: − 5.0 mL min^−1^ kg^−1^; men HUNT2: − 1.8 mL min^−1^ kg^−1^; men HUNT3: − 4.0 mL min^−1^ kg^−1^) (*p* < 0.001 for all comparisons). Online Resource 1 provides further details regarding eCRF in RA patients and controls in 10-year categories for both sexes.

### Sensitivity analyses for method validation

**Fig. 3:**
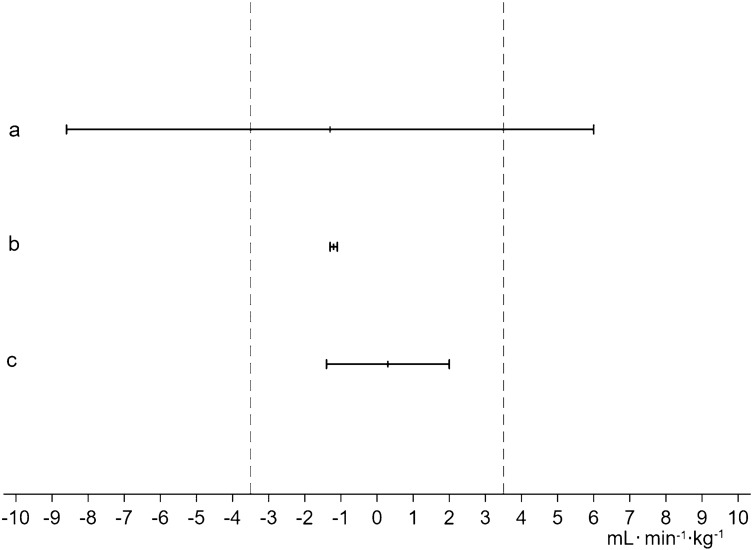
Equivalence testing for method validation. Methods are regarded equivalent when the 90% confidence interval (CI) of the difference between measurement with the two methods (horizontal bars) lie within the equivalence region (vertical dashed lines), defined as ± 1 MET (± 3.5 mL min^−1^ kg^−1^). Bar a: RA^d^ -specific equation used for healthy participants in HUNT3 Fitness compared to measured CRF. Mean difference: − 1.3 min^−1^ kg^−^1, 90% CI − 8.6, 6.0 mL· min^−1^ kg^−1^. Methods were non-equivalent. Bar b: RA-specific equation for HUNT3 compared to RA-specific equation for HUNT2. Mean difference: − 1.2 mL kg^−1^ min^−1^, 90% CI − 1.3, − 1.1 mL kg^−1^ min^−1^. Methods were equivalent. Bar c: General eCRF equation for HUNT3 compared to general eCRF equation for HUNT2. Mean difference: 0.3 mL min^−1^ kg^−1^, 90% CI − 1.4, 2.0 mL min^−1^ kg^−1^. Methods were equivalent. ^d^*RA* rheumatoid arthritis, *HUNT3 Fitness* Sub-study of HUNT3, *CRF* cardiorespiratory fitness, *HUNT2 and HUNT3* The second and third surveys of the Trøndelag Health Study, *eCRF* estimated cardiorespiratory fitness

The RA-specific equation was non-equivalent with measured CRF when used for healthy persons, confirming that eCRF in controls and RA patients cannot be calculated using the same equation (Fig. 3). The RA equations for HUNT2 and HUNT3 were equivalent, and so were the general eCRF equations for HUNT2 and HUNT3, demonstrating that change in eCRF from HUNT2 to HUNT 3 was not biased by the use of slightly different equations (Fig. 3).

## Discussion

Having an RA diagnosis was associated with a faster age-related decline in eCRF compared to controls, and this effect was larger with higher age at baseline. RA patients also had lower eCRF than controls, especially in the older age categories.

There has been much focus upon the fact that RA patients have worse cardiovascular risk factor profiles, excess CVD and excess mortality from CVD compared to the general population [3]. In theory, the faster decline in CRF associated with RA might be explained by their less favorable cardiovascular risk factors and higher incidence of CVD at an earlier age, contributing to a vicious cycle. In this study, women with RA had higher BMI and more often had hypertension compared to controls; whereas, more men with RA more often were ever smokers, had diabetes, CVD, or hypertension compared to controls. However, by adjusting for known risk factors for cardiovascular disease like BMI, smoking, and lower HDL cholesterol in addition to hypertension, asthma, and previous CVD, the faster decline in eCRF of RA patients compared to controls became even more pronounced, indicating that other factors were also involved. The association between CVD, risk factors, and CRF substantiates the importance of CRF improvement as a preventive measure of CVD in RA patients. The findings that fewer RA patients met the general recommendations for aerobic PA compared to controls and that fewer participants met the recommendations in HUNT3 than in HUNT2 are important from this perspective because the level of PA is a well-known predictor of CRF.

It could be important for interpretation of the results that advice and information about PA given to RA patients have changed in recent years. Advice recommending exercise with low intensity has gradually shifted towards advice about high-intensity exercise. Thus, more recent exercise regimens for RA patients could potentially have counteracted the decline in eCRF from HUNT2 to HUNT3. This does not seem to have had a strong effect because a study from our group showed that RA patients tested in 2017 still had reduced CRF compared to the healthy population [16].

For better care and follow-up of the general population, the AHA has recommended use of estimation models for eCRF [7], and the ACSM/AHA recommendations for PA are implemented as important aims for the level of physical activity in RA patients [23, 26]. However, development and implementation of suitable exercise programs for RA patients still need higher priority. Estimating CRF in RA patients can contribute to better follow-up. To facilitate correct estimation of eCRF, we have recently published equations that are customized for RA patients [15]. Uptake of these formulae in rheumatology practice may contribute to better patient care.

The proportion of healthy controls that fulfill the recommendations for PA has not changed much over the years. On the contrary, there is a trend of major concern for public health that inactivity at work has increased. Analyzing the effect of type of work (physical vs. non-physical) could be of interest in the present study as well, but due to missing data, this variable could not be included.

Other possible explanations for increased deterioration of eCRF by time in RA patients need to be considered. The natural process of aging contributes to deterioration of CRF by time. As RA is associated with accelerated aging of the immune system, including insufficiency of telomerase activity and deficiency of DNA repair mechanisms [27], one may speculate that such mechanisms contributed to the faster decline in eCRF. Further, RA is associated with rheumatoid cachexia, with reduced muscle mass and increased fat mass [6], which adds to the natural wasting of musculature by increasing age. This may render RA patients more susceptible to the frailty syndrome. An individual is considered frail if three out of these five phenotypes are present: weakness, unintentional weight loss, exhaustion, low PA and slower walking speed [28]. Frail persons have an increased frequency of negative health outcomes, including accidental falls, reduced mobility and decreased functional capacity [27]. Frailty could potentially contribute to reduced eCRF in RA patients, but unfortunately, we did not have data to assess frailty in the present study.

The present study has several strengths. It was population-based and included a substantial number of participants with ~ 11 years of follow-up. Furthermore, the RA diagnoses were validated from information in hospital case files [20]. A potential weakness is that eCRF for controls and RA patients were calculated using different equations, but the sensitivity analyses clearly showed that this did not bias the results. Our study confirmed that the RA-specific equations should only be used in RA patients. Another study from our group showed that the general eCRF equation is not adequate for RA patients because of a tendency towards underestimation in RA patients at highest risk of CVD [15]. The equations used in HUNT2 and HUNT3 were equivalent, both for RA patients and healthy controls. Taken together, our study supports that eCRF in RA patients and the general population should be calculated using different equations.

Because HUNT is a large population-based study, RA disease-related variables that would not be relevant for controls such as disease activity, swollen and tender joint counts, or the patient’s global disease assessment were not collected. Previously, our group found that a number of variables describing physical function and disease activity were not associated with measured CRF at CPET in RA patients and did not improve the RA-specific eCRF equation [15, 16]. Thus, the results of the present study are probably not biased because such variables were missing.

Since HUNT3 was performed in 2006–2008, there has been a change in treatment strategies for RA with more medications to choose from and use of higher doses of anti-rheumatic drugs like methotrexate. Thus, results from this study might not be representative for today’s RA population. In a former study [16], we investigated various predictors for the measured CRF in RA patients. Disease modifying anti-rheumatic drugs, comorbidities, and disease activity scores other than the patient global assessment were not significant predictors for CRF. These findings support that despite changes in treatment strategies the start of the present study, the results may still be representative.

A limitation of this study may be the use of estimation models for CRF instead of direct measurement. CPET of all participants would not easily be feasible in a study as large as HUNT, but a smaller future study using CPET could provide more accurate data. The low number of RA patients may represent a limitation, but the very large control group reduces selection bias and thereby improves the validity of the results.

In conclusion, the present study showed that age-related eCRF deterioration was faster in RA patients compared to healthy controls. This finding may add to the explanation of the increased frequency of CVD in RA patients at an earlier age compared to healthy controls. The study also found that a lower percentage of RA patients fulfilled recognized PA recommendations, and that RA patients had lower CRF at baseline. Thus, increasing PA in RA patients seems to be an important measure to improve cardiovascular health by reducing the age-related decline in eCRF, in addition to modern medical treatment.

## Electronic supplementary material

Below is the link to the electronic supplementary material.

Supplementary file1 (PDF 87 kb)

## Data Availability

Data from HUNT are available upon reasonable request from the HUNT Research Centre (www.ntnu.edu/hunt/data), following approval from the Regional Research Ethics Committee. However, restrictions apply to the availability of the data for the present paper, which were used under license for the current study and are not publicly available in accordance with Norwegian law.

## References

[1] Kourilovitch M, Galarza-Maldonado C, Ortiz-Prado E (2014). Diagnosis and classification of rheumatoid arthritis. J Autoimmun.

[2] Sokka T, Abelson B, Pincus T (2008). Mortality in rheumatoid arthritis: 2008 update. Clin Exp Rheumatol.

[3] Widdifield J, Paterson JM, Huang A, Bernatsky S (2018). Causes of death in rheumatoid arthritis: how do they compare to the general population?. Arthritis Care Res (Hoboken).

[4] Avina-Zubieta JA, Thomas J, Sadatsafavi M, Lehman AJ, Lacaille D (2012). Risk of incident cardiovascular events in patients with rheumatoid arthritis: a meta-analysis of observational studies. Ann Rheum Dis.

[5] van den Hoek J, Boshuizen HC, Roorda LD, Tijhuis GJ, Nurmohamed MT, van den Bos GA, Dekker J (2017). Mortality in patients with rheumatoid arthritis: a 15-year prospective cohort study. Rheumatol Int.

[6] Urman A, Taklalsingh N, Sorrento C, McFarlane IM (2018). Inflammation beyond the joints: rheumatoid arthritis and cardiovascular disease. Sci Fed J Cardiol.

[7] Ross R, Blair SN, Arena R, Church TS, Despres JP, Franklin BA, Haskell WL, Kaminsky LA, Levine BD, Lavie CJ, Myers J, Niebauer J, Sallis R, Sawada SS, Sui X, Wisloff U (2016). Importance of assessing cardiorespiratory fitness in clinical practice: a case for fitness as a clinical vital sign: a scientific statement from the American Heart Association. Circulation.

[8] Weston KS, Wisløff U, Coombes JS (2014). High-intensity interval training in patients with lifestyle-induced cardiometabolic disease: a systematic review and meta-analysis. Br J Sports Med.

[9] Myers J, McAuley P, Lavie CJ, Despres JP, Arena R, Kokkinos P (2015). Physical activity and cardiorespiratory fitness as major markers of cardiovascular risk: their independent and interwoven importance to health status. Prog Cardiovasc Dis.

[10] Nauman J, Nes BM, Lavie CJ, Jackson AS, Sui X, Coombes JS, Blair SN, Wisloff U (2017). Prediction of cardiovascular mortality by estimated cardiorespiratory fitness independent of traditional risk factors: the HUNT study. Mayo Clin Proc.

[11] Nes BM, Janszky I, Vatten LJ, Nilsen TI, Aspenes ST, Wisloff U (2011). Estimating V.O 2peak from a nonexercise prediction model: the HUNT Study. Nor Med Sci Sports Exerc.

[12] Krokstad S, Langhammer A, Hveem K, Holmen TL, Midthjell K, Stene TR, Bratberg G, Heggland J, Holmen J (2013). Cohort profile: the HUNT Study, Norway. Int J Epidemiol.

[13] Nes BM, Vatten LJ, Nauman J, Janszky I, Wisloff U (2014). A simple nonexercise model of cardiorespiratory fitness predicts long-term mortality. Med Sci Sports Exerc.

[14] Shigdel R, Dalen H, Sui X, Lavie CJ, Wisløff U, Ernstsen L (2019). Cardiorespiratory fitness and the risk of first acute myocardial infarction: the HUNT Study. J Am Heart Assoc.

[15] Liff MH, Hoff M, Fremo T, Wisloff U, Videm V (2020). An Estimation model for cardiorespiratory fitness in adults with rheumatoid arthritis. Med Sci Sports Exerc.

[16] Liff MH, Hoff M, Fremo T, Wisløff U, Thomas R, Videm V (2019). Cardiorespiratory fitness in patients with rheumatoid arthritis is associated with the patient global assessment but not with objective measurements of disease activity. RMD Open.

[17] Metsios GS, Koutedakis Y, Veldhuijzen van Zanten JJ, Stavropoulos-Kalinoglou A, Vitalis P, Duda JL, Ntoumanis N, Rouse PC, Kitas GD (2015). Cardiorespiratory fitness levels and their association with cardiovascular profile in patients with rheumatoid arthritis: a cross-sectional study. Rheumatology (Oxford).

[18] Munsterman T, Takken T, Wittink H (2012). Are persons with rheumatoid arthritis deconditioned? a review of physical activity and aerobic capacity. BMC Musculoskelet Disord.

[19] Loe H, Steinshamn S, Wisloff U (2014). Cardio-respiratory reference data in 4631 healthy men and women 20–90 years: the HUNT 3 fitness study. PLoS ONE.

[20] Videm V, Thomas R, Brown MA, Hoff M (2017). Self-reported Diagnosis of rheumatoid arthritis or ankylosing spondylitis has low accuracy: data from the Nord-Trondelag Health Study. J Rheumatol.

[21] Arnett FC, Edworthy SM, Bloch DA, McShane DJ, Fries JF, Cooper NS, Healey LA, Kaplan SR, Liang MH, Luthra HS (1988). The American Rheumatism Association 1987 revised criteria for the classification of rheumatoid arthritis. Arthritis Rheum.

[22] Aletaha D, Neogi T, Silman AJ, Funovits J, Felson DT, Bingham CO, Birnbaum NS, Burmester GR, Bykerk VP, Cohen MD (2010). 2010 Rheumatoid arthritis classification criteria: an American College of Rheumatology/European League Against Rheumatism collaborative initiative. Ann Rheum Dis.

[23] Garber CE, Blissmer B, Deschenes MR, Franklin BA, Lamonte MJ, Lee IM, Nieman DC, Swain DP (2011). Quantity and quality of exercise for developing and maintaining cardiorespiratory, musculoskeletal, and neuromotor fitness in apparently healthy adults: guidance for prescribing exercise. Med Sci Sports Exerc.

[24] Tibshirani R (1996). Regression shrinkage and selection via the lasso. J Roy Stat Soc.

[25] Dixon PM, Saint-Maurice PF, Kim Y, Hibbing P, Bai Y, Welk GJ (2018). A Primer on the use of equivalence testing for evaluating measurement agreement. Med Sci Sports Exerc.

[26] Rausch Osthoff AK, Niedermann K, Braun J, Adams J, Brodin N, Dagfinrud H, Duruoz T, Esbensen BA, Gunther KP, Hurkmans E (2018). 2018 EULAR recommendations for physical activity in people with inflammatory arthritis and osteoarthritis. Ann Rheum Dis.

[27] Boots AM, Maier AB, Stinissen P, Masson P, Lories RJ, De Keyser F (2013). The influence of ageing on the development and management of rheumatoid arthritis. Nat Rev Rheumatol.

[28] Fried LP, Tangen CM, Walston J, Newman AB, Hirsch C, Gottdiener J, Seeman T, Tracy R, Kop WJ, Burke G, McBurnie MA (2001). Frailty in older adults: evidence for a phenotype. J Gerantol.

