# Supplementary material for: Faster age-related decline in cardiorespiratory fitness in rheumatoid arthritis patients: an observational study in the Trøndelag Health Study
Source: Rheumatol Int. 2020 Oct 9;41(2):369–79. doi: 10.1007/s00296-020-04713-2 (PMC7835174; doi:10.1007/s00296-020-04713-2)
Supplement: Supplementary file 1 — Supplementary file1 (PDF 87 kb) [file 296_2020_4713_MOESM1_ESM.pdf]

Title: Faster age-related decline in cardiorespiratory fitness in rheumatoid arthritis patients – an observational study in the population-based Trøndelag Health Study

Authors: Marthe Halsan Liff, Mari Hoff, Ulrik Wisløff and Vibeke Videm

Corresponding author: Vibeke Videm, Department of Clinical and Molecular Medicine, NTNU – Norwegian University of Science and Technology and Department of Immunology and Transfusion Medicine, St. Olavs University Hospital, Trondheim, Norway. E-mail: vibeke.videm@ntnu.no

Online Resource 1: Estimated cardiorespiratory fitness (eCRF) in controls and rheumatoid arthritis (RA) patients in sex and 10-year age categories (30-89 years) in HUNT2 and HUNT3

	**Women**			**Men**		
**HUNT2**	**Controls, eCRF[a]**	**RA patients, eCRF[b]**		**Controls, eCRF[a]**	**RA patients, eCRF[b]**	
**Age**	**Mean eCRF (CI), n**	**Mean eCRF (CI), n**	***p*-value**	**Mean eCRF (CI), n**	**Mean eCRF (CI), n**	***p*-value**
30-89 years	33.9(33.8-33.9), 24,033	29.1(28.2-30.0), 253	<0.001	43.2(43.1-43.3), 23,103	38.9(37.4-40.3), 129	<0.001
30-39 years	40.3(40.2-40.4), 5,623	38.4(36.4-40.2), 22	0.01	50.3(50.2-50.5), 5,134	55.9(46.6-55.4), 6	<0.01
40-49 years	36.6(36.5-36.7), 6,496	35.4(34.4-36.5), 68	0.01	46.2(46.1-46.3), 6,104	48.6(46.8-50.3), 20	0.026
50-59 years	32.9(32.8-33.1), 4,994	28.5(27.5-29.4), 69	<0.001	42.2(42.0-42.3), 4,910	40.3(39.0-41.6), 46	<0.01
60-69 years	29.0(28.9-29.1), 3,441	23.9(22.7-25.1), 60	<0.001	38.3(38.2-38.5), 3,600	34.8(33.2-36.3), 37	<0.001
70-79 years	25.3(25.2-25.5), 2,608	21.3(19.8-22.9), 30	<0.001	34.7(34.5-34.9), 2,678	28.2(26.5-30.0), 20	<0.001
80-89 years	22.1(21.9-22.4), 871	18.6(11.3-25.9), 4	0.06	30.4(30.0-30.8), 677	n=0	
**HUNT3**	**Controls, eCRF[c]**	**RA patients, eCRF[d]**		**Controls, eCRF[c]**	**RA patients, eCRF[d]**	
**Age**	**Mean eCRF (CI), n**	**Mean eCRF (CI), n**	***p*-value**	**Mean eCRF (CI), n**	**Mean eCRF (CI), n**	***p*-value**
30-89 years	31.4(31.3-31.4), 20,169	23.5(22.5-24.5), 149	<0.001	39.2(39.0-39.3), 16,249	31.2(29.4-32.9), 85	<0.001
30-39 years	37.1(36.9-37.2), 3,189	28.1(5.9-50.2), 3	<0.001	46.6(46.4-46.9), 2,069	49.1, 1	NA
40-49 years	34.8(34.7-34.9), 4,668	33.3(31.1-35.4), 7	0.34	43.3(43.1-43.5), 3,406	48.3, 1	NA
50-59 years	31.5(31.4-31.7), 4,994	28.0(26.6-29.4), 47	<0.001	39.5(39.4-39.7), 4,153	41.2(39.0-43.4), 15	0.22
60-69 years	28.2(28.1-28.4), 4,222	23.3(22.0-24.5), 47	<0.001	36.2(36.0-36.3), 3,764	31.2(29.1-33.2), 32	<0.001
70-79 years	24.9(24.8-25.1), 2,284	18.2(17.0-19.3), 31	<0.001	33.0(32.8-33.2), 2,149	27.8(26.0-29.7), 28	<0.001
80-89 years	22.6(22.3-22.8), 812	15.1(13.5-16.8), 14	<0.001	29.7(29.4-30.1), 708	19.7(16.8-22.6), 8	<0.001

eCRF of controls, estimated by [a)]general eCRF formula developed for HUNT2 [1] and [c)]general eCRF formula developed for HUNT3 [2], compared to eCRF of RA patients calculated by [b)]RA-specific formula developed for HUNT2, and [d)]RA-specific formula developed for HUNT3 [3]. Rows in gray when n<6.

1. Nauman J, Nes BM, Lavie CJ, Jackson AS, Sui X, Coombes JS, Blair SN, Wisloff U (2017) Prediction of cardiovascular mortality by estimated cardiorespiratory fitness independent of traditional risk factors: the HUNT study. Mayo Clin Proc 92 (2):218-227.
2. Nes BM, Janszky I, Vatten LJ, Nilsen TI, Aspenes ST, Wisloff U (2011) Estimating V.O 2peak from a nonexercise prediction model: the HUNT study, Norway. Med Sci Sports Exerc 43 (11):2024-2030.
3. Liff MH, Hoff M, Fremo T, Wisløff U, Videm V (2020) An Estimation Model for Cardiorespiratory Fitness in Adults with Rheumatoid Arthritis. Med Sci Sports Exerc 52 (6):1248-1255.
